# Use of a chlorhexidine-impregnated dressing reduced catheter-related bloodstream infections caused by Gram-positive microorganisms

**DOI:** 10.12669/pjms.342.14810

**Published:** 2018

**Authors:** Ayse Betul Ergul, Ikbal Gokcek, Alper Ozcan, Serife Cetin, Nurkan Gultekin, Yasemin Altuner Torun

**Affiliations:** 1 Ayse Betul Ergul, M.D. Department of Pediatric Intensive Care Unit, University of Health Sciences, Kayseri Training and Research Hospital, Kayseri, Turkey; 2 Ikbal Gokcek, M.D. Department of Pediatric Intensive Care Unit, University of Health Sciences, Kayseri Training and Research Hospital, Kayseri, Turkey; 3 Alper Ozcan, M.D. Department of Pediatric Intensive Care Unit, University of Health Sciences, Kayseri Training and Research Hospital, Kayseri, Turkey; 4 Nurkan Gultekin, Nurse, Department of Pediatric Intensive Care Unit, University of Health Sciences, Kayseri Training and Research Hospital, Kayseri, Turkey; 5 Serife Cetin, Nurse, Department of Infection Control, University of Health Sciences, Kayseri Training and Research Hospital, Kayseri, Turkey; 6 Dr. Yasemin Altuner Torun, Associate, Professor. Department of Pediatric Hematology, University of Health Sciences, Kayseri Training and Research Hospital, Kayseri, Turkey

**Keywords:** Catheter-related bloodstream infection, Catheter securement, Chlorhexidine dressing, Intensive care

## Abstract

**Objective:**

We compared the protective effects of secure Chlorhexidine Gluconate (CHG)-containing dressings with those of non-antimicrobial transparent dressings.

**Methods:**

This prospective, comparative, single-center clinical study was conducted in a tertiary pediatric intensive care unit from October 2014 to March 2017. The inclusion criterion was catheterization of the jugular vein for ≥48 hour. The study was conducted in two phases. Non-antimicrobial standard dressings were applied both before and after the CHG- dressing phase to negate any coincidental temporal effect. During the standard-dressing phases, the dressings did not include any antimicrobial; transparent CHG-impregnated dressings were applied during the test phase. All patients were divided into two groups by the type of dressing applied (standard and CHG-containing dressings).

**Results:**

The standard- and CHG-dressing groups contained 68 and 63 patients, respectively. The median durations of catheterization were 13 (8–22) and 14 (2–28) days, respectively (*p*>0.05). The Catheter-Related Bloodstream Infection (CRBSI) rate was somewhat lower in the CHG-dressing group (20.6 vs. 26.5%), but the difference was not statistically significant (*p*>0.05). In the CHG-dressing group, CRBSIs caused by Gram-positive microorganisms totaled 0%, but the figure was 8.8% in the control group (*p*=0.028).

**Conclusions:**

CHG dressings reduced CRBSIs caused by Gram-positive microorganisms.

## INTRODUCTION

Central venous catheterization is common in Intensive Care Units (ICUs). Catheter-Related Bloodstream Infections (CRBSIs) are frequent complications of catheterization.^1^ In the USA, approximately 80,000 CRBSIs develop annually in ICUs, with mortality rates of up to 35%.^2^ Several strategies are employed to reduce CRBSIs; these include skin antisepsis, prescription of prophylactic antibiotics, the use of antimicrobial catheters, implementation of catheter care bundles, chlorhexidine baths, and addition of antimicrobials (in the form of antimicrobial locks or dressings) to catheters.^2^

Chlorhexidine Gluconate (CHG) is frequently used to prevent CRBSIs; CHG in the soap used to bathe patients may reduce CRBSIs. CHG can also be used to sterilize the catheter site and wound dressings.^3^ A catheter dressing is a protective material applied to the site of catheter insertion with the principal aim of securing the catheter in place, and may consist of taped gauze or a transparent material. Catheter dressings that incorporate antiseptics or antibiotics are becoming more common; commercial dressings are available but can also be improvised within hospitals.^3^

The type of dressing used may affect the infection rate.^3^ Many studies have explored the relationships between CRBSI frequencies and dressing types. Dressings containing CHG may reduce the incidence of CRBSIs.^4^

We explored whether the regular use of a secure CHG-containing dressing (compared to a non-antimicrobial transparent dressing) to protect the catheters of ICU patients reduced the frequency of CRBSIs.

## METHODS

This was a prospective, comparative, single-center clinical study. It was performed between October 2014 and March 2017 in a tertiary care pediatric ICU. The study was conducted in two phases. Non-antimicrobial standard dressings were applied before and after the CHG-dressing phase to negate any coincidental temporal effect. Prior to this study, the non-antimicrobial “3M Tegaderm I.V. Dressing” was used for standard catheter care. We applied CHG dressings to half of all patients. Appropriate staff training was given prior to and during the changeover period. After the CHG-dressing phase, standard dressings were reintroduced. This study was approved by our local ethics committee (number: 2017/327). We found that a sample size of 61 patients per group would afford 80% power at the 5% significance level for the detection of a 20% difference between the two groups.

The primary outcome was whether the new CHG dressing reduced the CRBSI rate.

The secondary outcome was whether the CHG dressing affected the types of organisms causing CRBSIs.

### Inclusion criteria

All patients with catheters in the jugular vein for ≥48 hour.

### Exclusion criteria

Any existing BSI; known hypersensitivity to the study dressing or CHG per se; and/or eczema, rash, lesions, burns, or other skin conditions that might compromise skin integrity at the catheter insertion site; a hemodialysis catheter; combined jugular and femoral central venous catheters; and a catheter inserted prior to ICU admission.

### Catheterization procedure

All catheterizations were performed by ICU pediatricians. Sedation and analgesia were achieved using ketamine (1 mg/kg) or fentanyl (1 µg/kg), plus midazolam (0.1 mg/kg). Before intervention, the access site was sterilized with a CH/alcohol solution (>0.5% CH in alcohol). Non-tunneled, temporary central venous catheters (Seldicath; Plastimed, Saint Leu La Forêt, France) were used for all catheterizations. Double-lumen, 4-Fr, temporary central venous catheters were placed in patients of body weight <5 kg, and triple lumen 5- and 7-Fr catheters in patients weighing 5–20 kg and >20 kg, respectively.

### Catheter dressing procedure

After fixation with sutures, the catheter site was coated with a transparent polyurethane film dressing (Tegaderm; 3M, St. Paul, MO, USA) or a transparent dressing containing CHG and a hydrocolloid (Tegaderm CHG IV Securement Dressing; 3M). The latter dressing is an adhesive, semipermeable, transparent polyurethane film incorporating a transparent gel pad containing 2% (w/w) CHG; the former dressing is an adhesive, semipermeable, transparent polyurethane film containing no antimicrobial. Dressings without CHG were replaced at 2-day intervals or as needed; CHG-containing dressings were replaced every seven days or as needed.^5^

### CRBSIs

In patients catheterized for >2 days, a CRBSI was defined as one or more particular pathogens in one or more blood culture samples obtained between day three after catheterization and day one after removal of the catheter, along with a failure to identify any other source of infection, plus fever (>38°C), chills, and hypotension in a patient of any age or the presence of at least one of the following in patients aged <1 year: fever (>38°C), hypothermia (<36°C), apnea, or bradycardia in addition to a failure to link positive culture findings to any other source of infection.^6^

### Statistical analysis

All statistical analyses were performed with the aid of SPSS version 22.0 (IBM SPSS, Statistics for Windows; IBM Corp., Armonk, NY, USA). Frequency data are expressed as counts (with percentages). Non-parametric continuous variables are expressed as medians (with the 25th–75th percentiles). The Mann-Whitney U-test was used to compare non-parametric variables between the two groups. Categorical variables were compared with the aid of the chi-squared test. In all tests, a *p*-value <0.05 was considered statistically significant.

## RESULTS

The two groups did not differ significantly in terms of any of age, gender, underlying chronic disease, catheterization duration, number of catheter lumina, the side of catheterization, the vein used for catheterization, or the use (or not) of sonographic guidance during catheterization (Table-I). The median durations of catheterization in the standard- and CHG-dressing groups were 13 (8–22) and 14 (2–28) days, respectively.

**Table-I:** The demographic and clinical features of all patients.

Characteristics	Standard dressing group (n=68)	CHG-dressing group (n=63)	p value
Age, months	17.7 (7.9-34.9)	11 (5.9-25)	0.139*
Gender, female/male, n (%)	38 (55.9)/30 (44.1)	30 (44.1)/33 (52.4)	0.344**
Weight, kg, n (%)	9.3 (5.3-15)	6.1 (4-10)	0.139*
Underlying chronic disease, present, n (%)	54 (50)	54 (51.9)	0.343**
Length of ICU stay, days	79 (21-211)	117 (27-239)	0.384*
Indwelling duration, days	13 (8-22)	14 (2-28)	0.852*
Catheter lumena analysis, 2/3, (n)	38 (55.9)/30 (44.1)	45 (71.4)/18 (28.6)	0.065**
Sonographic guidance, used, n (%)	15 (22.1)	22 (34.9)	0.102**
Catheterization vein, jugular, (n/%)	86 (100)	68 (100)	
Body side, right/left, n (%)	50 (73.5)/18 (26.5)	55 (52.4)/8 (12.5)	0.139**

ICU (intensive care unit) Non-parametric data was presented as medians (25th –75th percentile).

* , Mann whitney-u test was used.

** , Chi-square test was used.

The CRBSI rates did not differ significantly between the groups (26.5% [n=18] in the control and 20.6 [n=13] in the test group). No CRBSIs caused by Gram-positive microorganisms were evident in the CHG-dressing group, but such infections caused 8.8% of all CRBSIs in the standard-dressing group (p=0.028). There was no significant difference between-group difference in the numbers of CRBSIs caused by either fungi or Gram-negative microorganisms (Table-II).

**Table-II:** Catheter-related bloodstream infections in the two groups.

	Standard dressing group (n=68)	CHG dressing group (n=63)	p value*
CR-BSI, present, n (%)	18 (26.5)	13 (20.6)	0.432
Gram positive, present, n (%)	6 (8.8)	0 (0)	0.028
Gram negative, present, n (%)	9 (13.2)	11 (17.5)	0.668
Fungi, present, n (%)	3 (4.4)	2 (3.2)	0.712

CR-BSI (Catheter related blood stream infection)

* , Chi-square test was used.

The types of microorganisms causing CRBSIs are listed in Table-III. Gram-negative microorganisms were the most common agents in both groups. No CRBSI caused by *Acinetobacter baumannii* was evident in the CHG-dressing group, but this bacterium caused 8.8% (n=5) of infections in the standard-dressing group (p=0.028; Table-III).

**Table-III:** Microorganisms causing catheter-related bloodstream infections in the two groups.

	Standard dressing group (n=68)	CHG-dressing group (n=63)
Coagulase negative staphylococcus, present, n (%)	5 (7.4)	0 (0)
Acinetobacter spp., present, n (%)	5 (7.4)	0 (0)
Klebsiella spp., present, n (%)	2 (2.9)	1 (1.6)
E. coli, present, n (%)	1 (1.5)	4 (6.3)
Pseudomonas spp., present, n (%)	2 (2.9)	1 (1.6)
Serratia spp., present, n (%)	0 (0)	3 (4.8)
Sphingomonas spp., present, n (%)	0 (0)	2 (3.2)
Candida spp., present, n (%)	3 (2.5)	2 (3.2)

## DISCUSSION

The CRBSI rate was reduced in the CHG-dressing group compared with that in the standard-dressing group, but the difference did not attain statistical significance. However, no CRBSI caused by a Gram-positive microorganism was noted in the former group. CRBSIs are common complications. The risk of infection is affected by the catheter placement site, being more common after femoral (and, to a lesser extent, internal jugular vein) catheterization than after subclavian vein catheterization.^7^ Thus, only patients with jugular vein catheters were included in the present study. The duration of catheterization is also a significant risk factor; CRBSI rates increase after six days of catheterization. An increase in the number of catheter lumina also increases the infection rate.^8^ We found no significant effect of either catheterization duration or the number of lumina in either group. In general, a dressing is applied during catheter insertion and is changed every 2–7 days based on the catheter material, or is kept in place until the time of catheter removal unless the dressing becomes damp, loose, or visibly soiled.^2^ CHG release increases over time to 7 days. Such sustained release may reduce the microbial load at the insertion site, thus decreasing the infection risk.^5^ Therefore, the dressing was changed weekly in the CHG-dressing group but every other day in the standard-dressing group.

Skin disinfection with CH and alcohol was recommended in 2011 by the Centers for Disease Control and Prevention (CDC) to prevent CRBSIs. We followed this recommendation for all patients. The same guidelines recommend the use of CHG- impregnated dressings.^9^ CHG suppresses bacterial growth at injection sites and reduces the incidence of CRBSIs. The antimicrobial activity of the CHG dressing is unique among existing dressings. During vascular access, CHG dressings are typically applied after skin preparation with antiseptics; the dressings form a waterproof barrier protecting against gross bacterial contamination. Although a non-antimicrobial dressing may minimize colonization, such a dressing cannot counter the regrowth of natural skin flora.^10^

CRBSI-related costs are reduced when CHG dressings are applied.^11^ We found that the CRBSI rate was lower in the CHG-dressing group than in the standard-dressing group, but statistical significance was not attained. Camins et al.^12^ studying hemodialysis patients with tunneled central venous catheters, found that the CRBSI rate was reduced when CHG dressings were applied. In pediatric patients undergoing central venous catheterization, Düzkaya et al.^13^ found that the CRBSI rate was lower in a CHG-dressing group than in a standard-dressing group but, again, statistical significance was not attained. The length of hospital stay, the duration of catheterization, and that of mechanical ventilation were shorter in the former than in the latter group; thus, the reduced CRBSI rate in the CHG-dressing group was considered to be clinically relevant.^13^ Although we found no significant between-group difference in the CRBSI rate, no CRBSI caused by a Gram-positive microorganism was noted in the CHG-dressing group.

Afonso et al. reported that nosocomial infections caused by skin flora decreased when CH-containing soaps were used.^14^ Mendes et al. also found that Vancomycin Resistant *Enterococcus* (VRE) colonization and infection rates fell when CH soaps were used for bathing, but the Gram-negative infection rate was not affected.^15^ Moreover, Petlin et al. found that the rate of infection with methicillin-resistant *Staphylococcus aureus* fell upon bathing with CH soap.^16^ We found that the CHG dressing prevented Gram-positive CRBSIs. CHG is effective against *A. baumannii* infections. Hayashi et al.^17^ also found that CH killed drug-resistant *Acinetobacter* strains. Our finding that no CRBSI caused by a Gram-positive microorganism was evident in the CHG-dressing group is significant.

Infection is also a common cause of catheter removal.^1^ In our study, a CRBSI was the second most common cause of removal (after elimination of the need for a catheter). No significant difference in terms of the causes of catheter removal was evident between the groups. The rate of accidental catheter removal did not differ significantly between the two groups, although the frequency of dressing changes did differ.

The principal limitation of our study is the small sample size; we may have lacked adequate power to detect a significance between-group difference in the CRBSI rate. Another limitation is the lack of randomization.

## CONCLUSION

The CHG dressing reduced CRBSIs caused by the Gram-positive microorganism in pediatric patients.
